# Projected impact of the Portuguese sugar-sweetened beverage tax on obesity incidence across different age groups: A modelling study

**DOI:** 10.1371/journal.pmed.1003036

**Published:** 2020-03-12

**Authors:** Francisco Goiana-da-Silva, Milton Severo, David Cruz e Silva, Maria João Gregório, Luke N. Allen, Magdalena Muc, Alexandre Morais Nunes, Duarte Torres, Marisa Miraldo, Hutan Ashrafian, Ana Rito, Kremlin Wickramasinghe, João Breda, Ara Darzi, Fernando Araújo, Carla Lopes

**Affiliations:** 1 Centre for Health Policy, Institute of Global Health Innovation, Imperial College London, London, United Kingdom; 2 Department of Public Health and Forensic Sciences and Medical Education, Faculty of Medicine, University of Porto, Porto, Portugal; 3 Epidemiology Research Unit, Institute of Public Health, University of Porto, Porto, Portugal; 4 Centre for Innovation, Technology and Policy Research, University of Lisbon, Lisbon, Portugal; 5 Directorate General of Health, Lisbon, Portugal; 6 Faculty of Nutrition and Food Sciences, University of Porto, Porto, Portugal; 7 Nuffield Department of Primary Care Health Sciences, University of Oxford, Oxford, England; 8 Department of Psychological Sciences, University of Liverpool, Liverpool, United Kingdom; 9 Centre for Public Administration and Public Policies, Institute of Social and Political Sciences, University of Lisbon, Lisbon, Portugal; 10 Department of Economics and Public Policy, Imperial College Business School, London, United Kingdom; 11 Centre for Health Economics and Policy Innovation, Imperial College Business School, London, United Kingdom; 12 Department of Surgery and Cancer, Imperial College London, London, United Kingdom; 13 WHO Collaborating Centre for Nutrition and Childhood Obesity, National Institute of Health (INSA), Lisbon, Portugal; 14 WHO European Office for the Prevention and Control of Noncommunicable Diseases, WHO Regional Office for Europe, Moscow, Russian Federation; 15 University Hospital of São João, Faculty of Medicine, University of Porto, Porto, Portugal; Carolina Population Center, UNITED STATES

## Abstract

**Background:**

Excessive consumption of sugar has a well-established link with obesity. Preliminary results show that a tax levied on sugar-sweetened beverages (SSBs) by the Portuguese government in 2017 led to a drop in sales and reformulation of these products. This study models the impact the market changes triggered by the tax levied on SSBs had on obesity incidence across various age groups in Portugal.

**Methods and findings:**

We performed a national market analysis and population-wide modelling study using market data for the years 2014–2018 from the Portuguese Association of Non-Alcoholic Drinks (GlobalData and Nielsen Consumer Panel), dietary data from a national survey (IAN-AF 2015–2016), and obesity incidence data from several cohort studies. Dietary energy density from SSBs was calculated by dividing the energy content (kcal/gram) of all SSBs by the total food consumption (in grams). We used the potential impact fraction (PIF) equation to model the projected impact of the tax-triggered change in sugar consumption on obesity incidence, through both volume reduction and reformulation. Results showed a reduction of 6.6 million litres of SSBs sold per year. Product reformulation led to a decrease in the average energy density of SSBs by 3.1 kcal/100 ml. This is estimated to have prevented around 40–78 cases of obesity per year between 2016 and 2018, with the biggest projected impact observed in adolescents 10 to <18 years old. The model shows that the implementation of this tax allowed for a 4 to 8 times larger projected impact against obesity than would be achieved though reformulation alone. The main limitation of this study is that the model we used includes data from various sources, which can result in biases—despite our efforts to mitigate them—related to the methodological differences between these sources.

**Conclusions:**

The tax triggered both a reduction in demand and product reformulation. These, together, can reduce obesity levels among frequent consumers of SSBs. Such taxation is an effective population-wide intervention. Reformulation alone, without the decrease in sales, would have had a far smaller effect on obesity incidence in the Portuguese population.

## Abstract 2

Francisco Goiana-da-Silva and colleagues invetigate the impact the market changes, triggered by the tax levied on sugar-sweetened beverages (SSBs), on obesity incidence across various age groups in Portugal.

## Author summary

**Why was this study done?:**

- There is high prevalence of obesity in many developed countries, including Portugal.
- Excessive consumption of sugar, and particularly soft drinks, has been linked with obesity.
- A previous study showed that a tax on sugar-sweetened beverages implemented by the Portuguese government in 2017 led to a reduction in sugar consumption.

**What did the researchers do and find?:**

- This study uses market data, dietary data, and obesity incidence data from several sources.
- This study models the impact of the Portuguese sugar-sweetened beverage tax policy on beverage reformulation and consumption, and assesses its consequent impact on obesity incidence across various age groups in Portugal.
- Results show a reduction of 6.6 million litres of soft drinks sold per year.

**What do these findings mean?:**

- The tax triggered a reduction in the demand for sugar-sweetened beverages.
- The tax triggered product reformulation (reduction of sugar content).
- The medium-term tax-related reduction in sugar consumption is estimated to prevent from 40 to 78 new cases of obesity in Portugal every year.
- The joint projected impact of product consumption reduction and product reformulation on obesity was 4 to 8 times larger than the projected impact of reformulation alone.

## Introduction

Obesity has reached epidemic levels in many developed countries including Portugal. Recent data show that 22.3% of Portuguese people are obese and 34.8% are pre-obese [1]. Portugal is the European country with the highest incidence of diabetes [2], and 4 in 10 children have excessive weight [3].

Excessive consumption of free sugars, and particularly sugar sweetened beverages (SSBs) has been linked with obesity and related adverse health outcomes [4–7]. The World Health Organization (WHO) recommends that free sugars not represent more than 10% of total daily energy intake [8]. Also, SSBs provide no nutritional value apart from hydration [4].

Therefore, the consumption of SSBs has been the target of several public health interventions. Evidence shows that taxing SSBs is an effective means of decreasing their consumption, and empirical and modelling studies link SSB taxation to a decrease in the incidence of obesity, especially among young people [9–11].

In Portugal, in 2013–2014, the per capita availability of sugar, obtained from Statistics Portugal Food Balance Sheet data, was 34.4 kg/year, which translates to 375 kcal per day from sugar [12]. According to the Food and Agriculture Organization of the United Nations [13], food balance sheets provide information on a country’s food system. In short, food balance sheets show trends in the national food supply, changes in the types of food consumed, and the extent to which food supply is in line with nutritional requirements. These data show that the per capita availability of sugar in Portugal is nearly twice as high as the WHO recommended maximum sugar intake, highlighting the urgent need for public health interventions. According to data from the National Food, Nutrition and Physical Activity Survey 2015–2016 (IAN-AF 2015–2016) [14], the individual consumption of added sugar was estimated to be 31.2 grams/day, and 24.4% of Portuguese people exceed the recommended daily sugar intake. Consumption levels are even more alarming among adolescents, as 51.1% of boys and 49.1% of girls in this age group exceed the recommended 200 kcal from sugar per day [14]. Consumption of SSBs in Portugal doubled between 1990 and 2012 [12], and in 2015–2016, 18% of all people and 42% of adolescents consumed over 220 g of SSBs and juices per day. Roughly 25% of regular consumers of these drinks report drinking an equivalent of 2 cans per day (330 ml each) [15]. Studies show that even very small children are regular consumers of soft drinks. A cohort study from Portugal showed that 35% of 2 year olds and 88% of 4 year olds drink SSBs weekly [16].

In response to high SSB consumption levels, and as a part of the State Budget for 2017, on 1 February 2017, the Portuguese government introduced a special consumption tax levied on sweetened beverages [17]. The so-called sugar tax allowed for a 2-month period of stock adjustment, during which previously stocked drinks were not taxed.

The levy covered all SSBs, referring to any drink with added sugar or other sweeteners, and excluded drinks considered as having nutritional value such as milk or “dairy alternative” beverages (e.g., soy, rice, oat, almond, hazelnut, coconut), fruit juices, and drinks considered food for special dietary needs or nutritional supplements. Alcoholic drinks were not included in the SSB taxation policy.

The taxable unit was hectolitres of final product sold, except for drink concentrates (for which the taxable unit was based on the sugar content of the reconstituted drink), and the tax was structured into 2 tiers: drinks with sugar content below 80 grams (based on total sugar) per litre were taxed at €8.22/hl, and those with sugar content of 80 grams or more per litre or more were taxed at €16.69/hl. The goal was to encourage reformulation by shifting drinks from the higher to the lower taxation tier [17].

Following the introduction of such a SSB tax, the reduction in sugar intake from SSBs results from 2 main processes: reduction of sugar available on the market, for example through reformulation, and decrease in demand for these drinks, driven by substitution effects due to increased prices as well as an increased awareness of their health impact. Preliminary results of the Portuguese sugar tax [18,19] showed that the introduction of the levy had an important impact on the SSB market, with a decrease in sales estimated at 7%. In addition, the reformulation of products towards lower levels of sugar, in order to avoid the higher tax, caused an estimated reduction of the energy intake from SSBs of 11%. Reformulation (reduced energy density) and decreased sales (reduced consumption) seem to have prevented or delayed 27 deaths caused by excessive consumption of sugar [20]. This paper aims to extend the analyses of the health benefits of the sugar tax by assessing its projected impact on obesity incidence.

A meta-analysis from 2016 [16] showed that the energy density from SSBs, measured as the dietary energy density (DED) from SSBs, defined as the ratio of SSB energy intake to total food consumed (in grams), is associated with elevated BMI (odds ratio [OR] 1.13, 95% CI 1.00–1.27) and with excess of adiposity (OR 1.27, 95% CI 1.04–1.55). It would be expected that the taxation of SSBs would lead to a decrease of the DED in the population. Since it is frequently argued that voluntary reformulation could lead to similar benefits, we also modelled the impact of reformulation alone on obesity incidence. This estimation does not account for any reduction in sales triggered by taxation. Nevertheless, it is important to note that, since SSB demand may be affected by price and product formula, it is difficult to totally disentangle the impact of each factor.

Population-wide interventions have been repeatedly shown to be more effective than those directed at individuals [21]; however, directly measuring the effect of population-wide interventions on health outcomes is challenging due to many cofounding factors. It is hard to separate the effect of taxation from other factors affecting obesity levels. Often, statistical modelling is used to assess this impact. One way of estimating the proportional decrease in obesity levels resulting from a specific population-level intervention is by calculating the potential impact fraction (PIF) [22]. We used PIF to quantify the prevented cases of obesity due to the reduction of DED.

### Objectives

In this study we aimed to estimate the impact of the Portuguese sugar tax on obesity incidence, across different age groups, due to product reformulation and sales (or consumption) reduction.

## Methods

### Data sources

We used several data sources: Nielsen Consumer Panel data from 2013 to 2018 (provided by the Portuguese Association of Non-Alcoholic Drinks [PROBEB]) containing sales of SSBs in the Portuguese market; tax revenue data from 2017 to 2018 provided by the Portuguese Ministry of Finance/Tax and Customs Authority; data on yearly sugar content of SSBs by product from GlobalData, provided by PROBEB; individual dietary consumption data from IAN-AF 2015–2016 [15,23]; and obesity incidence data by age group from several Portuguese cohorts, namely Generation XXI [24,25], EPITeen [26], and EPIPorto [27].

We used sales and tax revenue data to build a monthly sales panel. This information was then linked to individual dietary data and to SSB sugar content data to estimate the trend for DED. Finally, we estimated the impact of DED from SBBs on obesity incidence across different age groups.

### Trends of SSB sales and sugar content

To estimate the trend in sales of SSBs (in volume), we used retail sales data from the Nielsen Consumer Panel and data on SSB tax revenues from the Tax and Customs Authority.

The Nielsen Consumer Panel contains monthly retail sales data at the product level, namely, volume sold (litres) and total sales (€). The data are structured according to product market share, segment, category, carbonation of the drink, producer, brand, sub-brand, and flavour. The Nielsen Consumer Panel does not include demographic information on buyers of specific products as it only reports aggregate sales of specific items.

The sample used in the analyses covers the years from 2013 to 2018 for all private label brands (i.e., excluding white label brands), which constitutes around 67% of the Portuguese SSB market share. (White labelling is when a company removes its branding from the end product and uses the branding requested by the purchaser/retailer.) This included 125 commercial brands and 5,038 products. The Nielsen Consumer Panel registers the sales of 100% of hypermarkets (100/100), 96% of big supermarkets (410/427), 89% of small supermarkets (1,013/1,130), approximately 3% of traditional local stores, and 0.76% of hotels, restaurants, and coffee shops (550/72,538) in Portugal.

Albeit more detailed than the tax revenue data, the Nielsen data cover only 67% of the market. Therefore, we also used data from tax revenues that, albeit at an aggregated level, cover the entirety of sales of SSBs in the Portuguese market. These data consist of monthly tax revenues per taxation tier (beverages with 80 g of added sugar per litre or more and beverages with less than 80 g of added sugar per litre). These data were used to calibrate the Nielsen data. Since the tax was only introduced in January 2017, the tax revenue data are only available from that date to 2018, and therefore they could not be used to perform the whole analyses.

To estimate the trend of the sugar content of SSBs, the sales data were matched to sugar content data from GlobalData.

The GlobalData dataset contains the sugar and energy content evolution data for each SSB brand, sub-brand, and flavour identified in the Nielsen retail sales data. This sugar content evolution covers the years from 2013 to 2018 and is communicated directly by each SSB producer to GlobalData.

Data on SSB sugar and energy content (grams/100 ml) from GlobalData (from 2016) were merged with the SSB consumption of each individual, from the IAN-AF 2015–2016 [15,23], by brand and flavour descriptors.

The data from IAN-AF 2015–2016 were used to define the baseline of total food consumption and, specifically, consumption of SSBs and corresponding information on the average sugar and energy intake from SSBs. Briefly, the survey includes a representative sample of the Portuguese general population from 3 months to 84 years of age, selected from the national health registry by multistage sampling. Complete dietary data were collected in 5,811 individuals, using two 24-hour recalls (8 to 15 days apart) and also complemented by a food propensity questionnaire. Then, building on the baseline, the trend in average sugar and energy intake from SSBs was estimated considering the percentage reduction observed in retail sales data or in sugar content (GlobalData), in the following years.

The total amount of food consumed by each individual was used to estimate the baseline DED, by age groups.

### Obesity incidence

Data from Portuguese cohorts across different age groups among children (Generation XXI) [24,25], adolescents (EPITeen) [26], and adults (EPIPorto) [27] were used to estimate obesity incidence. Generation XXI is a birth cohort that includes a total of 8,647 live newborns enrolled between April 2005 and August 2006 at public maternity units from the metropolitan area of Porto, Portugal. EPITeen is a population-based cohort of adolescents, comprising those born in 1990 and attending public and private schools in Porto, Portugal, during 2003–2004; 2,159 adolescents who were 13 years old at baseline were recruited. The EPIPorto cohort included 2,485 adults between the ages of 18 and 90 years at baseline living in the city of Porto, recruited in 1999–2003 by random digit dialling.

### Data analysis

In order to measure the impact of the sugar tax on obesity incidence, we first estimated its impact on consumption (proxied by sales). Estimated consumption levels were then used to calculate the DED evolution from the baseline year of 2016 (pre-taxation) until 2018 (post-taxation).

In order to do so, we set out to obtain data on individual dietary intake (sugar and energy from SSBs and total amount of food consumption) to estimate the DED, trends in SSB consumption proxied by market data, the association between DED and obesity (extracted from the literature), and obesity incidence across different age groups. This allowed us to estimate the individual DED, the PIF, and the number of cases prevented in 3 counterfactual scenarios.

Baseline DED from SSBs was calculated, at the individual level, considering the energy content (kcal/gram) of all SSBs consumed by an individual divided by the amount of total food that same individual consumed (in grams), using information from the IAN-AF 2015–2016 [15,23].

$${DED}_{i}=\frac{{SSB\;energy\;intake}_{i}}{{total\;amount\;of\;food\;consumption}_{i}}$$

This estimate considered the consumption of SSBs by brand, and for the beverages with missing information of brand (18.9%), multiple imputation was performed and the pooled prevalence of the 5 generated numbers was considered. The imputed values were generated according to the prevalence of brands within each type of beverage. The rules of Rubin [28] were used for combining the prevalence of each brand from each imputed dataset into the overall multiple imputation estimate and the respective associated standard error.

The first counterfactual scenario only takes into account reduction in sugar content due to product reformulation in 2017, maintaining constant the volume consumed according to the baseline information on individual consumption (IAN-AF 2015–2016). The second counterfactual scenario includes changes in both sugar content and SSB sales volume based on the observed retail sales data, obtained from the Neilson Consumer Panel of total SSB sales calibrated with information from tax revenues, as described below. The third counterfactual scenario includes changes in both sugar content and SSB sales volume, but only uses data from tax revenues. The assumption of the researchers was that the first counterfactual gives the lowest value of potential impact of the taxation policy, the third counterfactual gives the highest potential impact of the taxation policy, and the second counterfactual is closest to the expected natural trend of consumption from the taxation policy.

Given that the sales volume of SSBs (*V*_*t*_) from both the Nielsen market and tax revenue data shows a seasonality effect, a trigonometric linear regression model was used to obtain the trend of SSB sales volume (model 1). This model was applied separately to tax revenue and Nielsen market data.

$$V_{td}=\beta_{0d}+\beta_{1d}Y_{t}+\beta_{2d}\sin\left(\frac{M_{t}-1}{12}2\pi \right)+\beta_{3d}\cos\left(\frac{M_{t}-1}{12}2\pi \right)+\epsilon$$

with *V*_*td*_ for data source *d =* representing the sales from the tax revenue data (*d = r*) and from the Nielsen market data (*d = m*).

The first and second terms of the equation allow for the estimation of the linear trend by year (β_0*d*_+β_1*d*_×*Y*_*t*_), while the third and fourth terms allow for the estimation of the seasonal trend by month s(*Md*_*t*_).

$$\left(s(M_{\mathrm{td}})=\beta_{2d}\sin\left(\frac{M_{t}-1}{12}2\pi \right)+\beta_{3d}\cos\left(\frac{M_{t}-1}{12}2\pi \right)\right)$$

For the missing tax data in the period pre-taxation, we assumed a linear projection using the estimated coefficients from model 1. Since the market data also are not complete for the year of 2018, also a linear projection was assumed for the period after 2017 using the estimated coefficients from model 1.

For the Nielsen market data, a calibration to represent the total volume was performed. The Nielsen market data only represent private label products, covering only 67% of the market, but allow for a more disaggregated analysis, whilst tax revenue data (also referred to as tax data) represent all SSBs, assuming that tax declaration and payment of taxes is accurate and complete.

The calibration of the Nielsen market data (${\hat{V}}_{tm}$) was estimated using the following model:

$$V_{tr}=\beta_{0c}+\beta_{1c}{\hat{V}}_{tm}+\epsilon \;(\mathrm{model}\;2)$$

$\hat{\beta_{0m}},\;\hat{\beta_{1m}},\;\hat{\beta_{0c},}$ and $\hat{\beta_{1c}}$ were used to calibrate the Nielsen market data in order to estimate the percentual change of SSB sales volume from the baseline pre-taxation year (2016) to the post-taxation year (2018), to be used in the counterfactual scenario 2 (2016–2018) (PPVSSB_2_):

$${PPVSSB}_{2}=\frac{{\hat{V}}_{tr=2018}}{{\hat{V}}_{tr=2016}}\times 100=\frac{\hat{\beta_{0c}}+\hat{\beta_{1c}}({\hat{V}}_{tm=2018})}{\hat{\beta_{0c}}+\hat{\beta_{1c}}({\hat{V}}_{tm=2016})}\times 100=\frac{\hat{\beta_{0c}}+\hat{\beta_{1c}}(\hat{\beta_{0m}}+\hat{\beta_{1m}}2018+s(Month=6))}{\hat{\beta_{0c}}+\hat{\beta_{1c}}(\hat{\beta_{0m}}+\hat{\beta_{1m}}2016+s(Month=6))}\times 100$$

The Sobel formula [28] was used to estimate the 95% confidence intervals (CIs) for the estimate of the decreased sales volume of SSBs, after calibrating ($\hat{\beta_{1c}}\times \hat{\beta_{1m}}$).

The estimated $\hat{\beta_{0r}}$ and $\hat{\beta_{1r}}$, from model 1, were used to estimate the percentage change of SSB sales volume using tax revenue information from baseline to 2018, to be used in the counterfactual scenario 3 (2016–2018) (PPVSSB_3_):

$${PPVSSB}_{3}=\frac{{\hat{V}}_{tr=2018}}{{\hat{V}}_{tr=2016}}\times 100=\frac{\left(\hat{\beta_{0r}}+\hat{\beta_{1r}}\times 2018+s(Month=6)\right)}{\left(\hat{\beta_{0r}}+\hat{\beta_{1r}}\times 2016+s(Month=6)\right)}\times 100$$

A linear projection was assumed for the period after 2017 using the estimated coefficients from model 1 and model 2.

The SSB individual intake was projected for counterfactual scenarios 2 and 3 by multiplying SSB individual intake over PPVSSB_2_ and PPVSSB_3_, respectively.

These projections were used to compute the SSB DED, as explained above. The SSB DED was divided into 6 categories according to the 5th, 25th, 50th, 75th, and 95th percentiles, obtained from the usual intake distribution (SPADE) [29], and was used to estimate the relative risk (RR) for each DED category, using the following formula:

RR(x)=exp(ln(a)×x)

where *a* is the association between DED and obesity extracted from a meta-analysis that combined data from 23 prospective cohort studies (*a* = 1.13) [30] and *x* is the value of DED for the upper limit of the category for all categories except the last, for which *x* was the lower limit plus the amplitude of the previous interval.

The estimated RRs were then used to compute the PIF using the RR shift formula:

$$PIF=\frac{\sum_{c=1}^{n}p_{c}{RR}_{c}-\sum_{c=1}^{n}p_{c}{RR}_{c}^{*}}{\sum_{c=1}^{n}p_{c}{RR}_{c}}$$

where ${RR}_{c}^{*}$ is the RR of category *c* for the counterfactual scenario of the dietary intensity associated with the consumption of SSBs, RR_*c*_ is the RR for category *c* for the baseline of SSB DED, and *p*_*c*_ is the proportion of the population in category *c*. The number of prevented cases is given by

$$n=PIF\times N\times I_{p}$$

where *N* is number of individuals in the population and *I*_*p*_ is the incidence of obesity in the population. The number of individuals in the population by age category was obtained from census data. Data on obesity incidence and 95% CIs were obtained from the referred Portuguese cohorts [24,26,27,31].

The estimates for the number of obesity cases prevented assume no further reformulations or changes than taxation of SSBs.

This study did not have a prospective study protocol. All analyses were carried out according to what was initially planned, and there were no data-driven changes to the analyses. The manuscript was revised in response to reviewers’ suggestions. However, the revisions did not change the nature nor the scope of the analyses.

## Results

**Fig 1:**
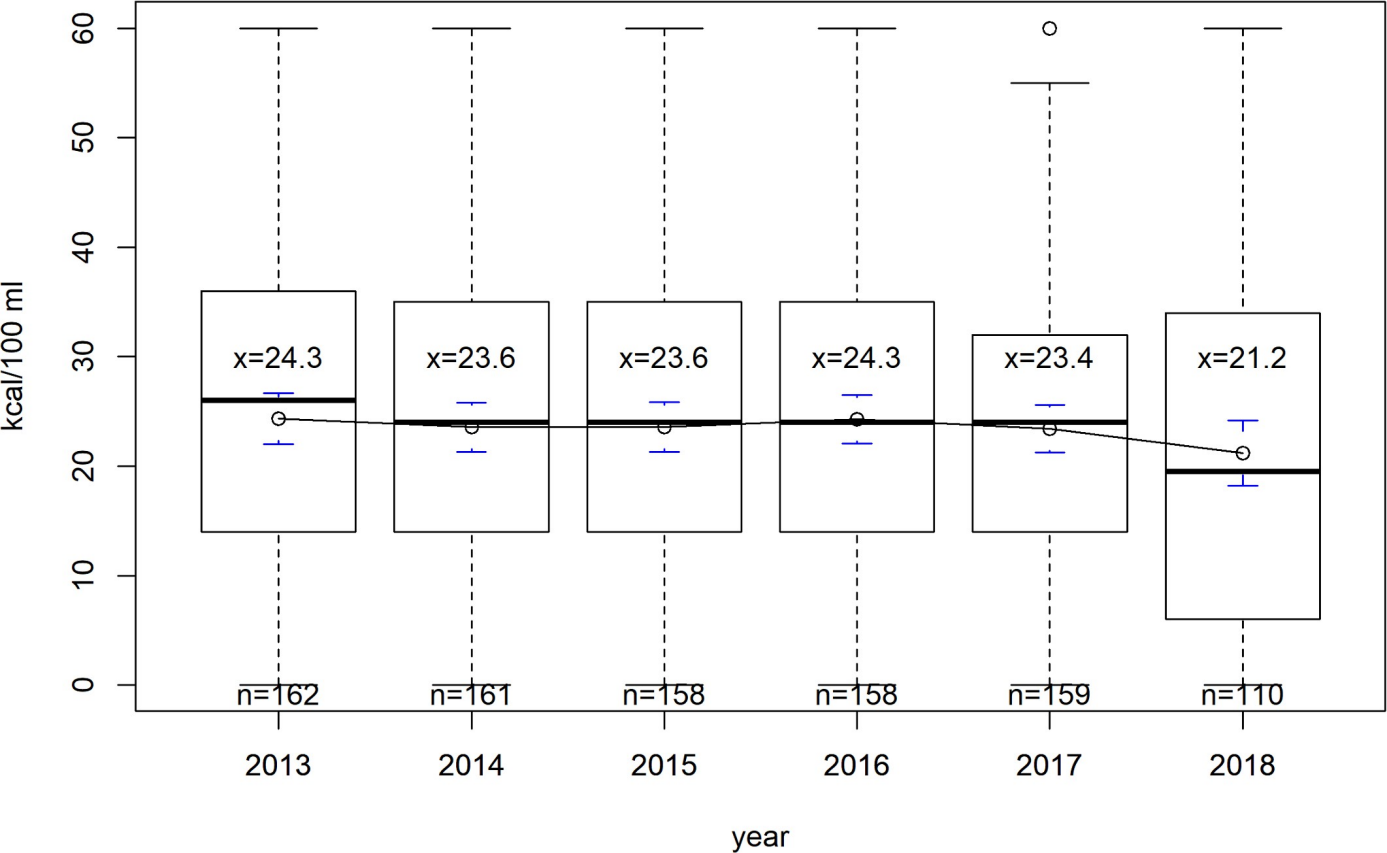
Time trend of the average energy intake from sugar-sweetened beverages (in kcal/100 ml) per sugar-sweetened beverage product, using market data.

In Fig 1 we can observe a significant quadratic trend of energy intake from SSBs (*p* < 0.001) by SSB product, using market data. The average estimates seem to have been quite stable before 2016 and decreased from 24.3 kcal/100 ml in 2016 to 21.2 kcal/100 ml in 2018. Applying a linear spline with a knot in 2017, we confirmed that the linear trend (in kcal/100 ml/year) observed until 2016 was not significant (β = −0.14, 95% CI −0.43 to 0.14), but from then on there is a significant linear decrease (β = −3.34, 95% CI −4.64 to −2.03).

**Fig 2:**
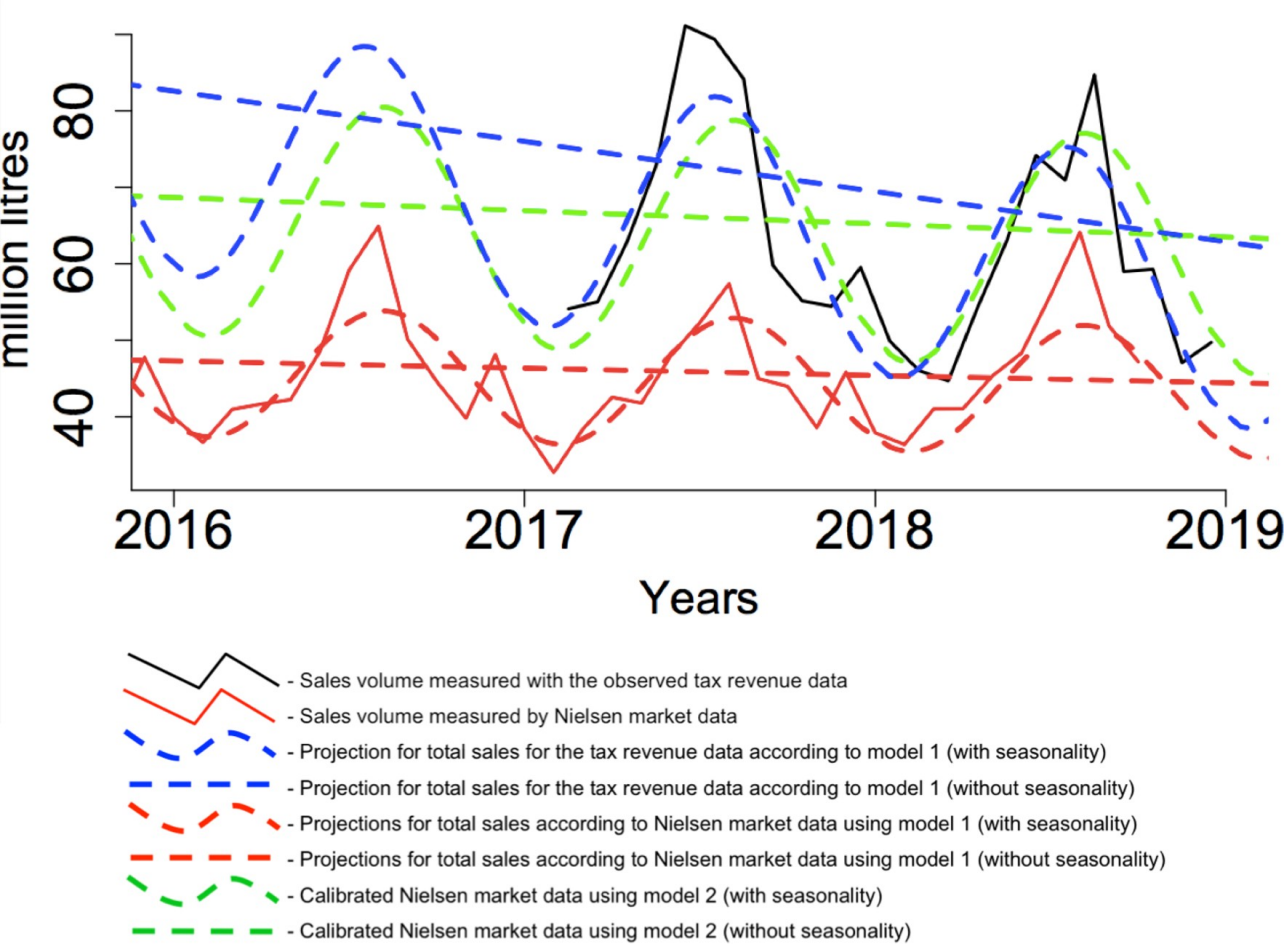
Time trend of total sales of sugar-sweetened beverages according to different data sources. Time trend of sales of sugar-sweetened beverages measured with the observed tax revenue data, only available post-tax (solid black line), and measured by Nielsen market data, available for the entire period 2016–2018 (red solid line); projections for total sales from the tax revenue data according to model 1 (dashed blue line with and without seasonality), projections for total sales according to market data using model 1 (dashed red line with and without seasonality); and calibrated Nielsen data from model 2 (dashed green line with and without seasonality). Dashed curves represent the data with seasonality, while straight lines represent the data without seasonality.

Fig 2 presents the time trend of the total sales of SSBs according to different data sources. First, we used the observed data from market sales (Nielsen Consumer Panel) from 2016–2018 (solid red line) and the corresponding values estimated by model 1 (dashed red line). Since the Nielsen Consumer Panel corresponds to only 67% of the market, data from tax revenues for 2017 and 2018 (solid black line) were used in model 1 in order to have a more accurate projection for the whole time period being analysed (dashed blue line). The second model includes calibration of market data to the total sales volume using tax information (dashed green line).

Since the observed time trend for SSB consumption showed strong seasonality (Fig 2), the final trends were adjusted. After adjusting for this effect, a decrease of 6.58 (95% CI 0.25 to 12.90) million litres per year was observed using tax data (linear blue line), while a decrease of 0.95 (95% CI 0.16 to 1.74) million litres per year was observed using market data (linear red line). Calibration of market data (model 2) showed a decrease of 1.73 (95% CI 0.20 to 3.25) million litres per year (linear green line). This corresponds to a percent decrease in consumption of 11% and 21% for market data and tax data, respectively, compared to the baseline consumption data of IAN-AF 2015–2016.

Table 1 presents the percentile distribution of DED from SSBs, by age group, in 2016 (baseline) and in 2018, considering the different counterfactual scenarios. The median DED at baseline was higher in adolescents (aged 10 to <18 years) (1.24%). Considering the different counterfactual scenarios, the estimates of DED from SSBs remain higher for adolescents but decrease as expected when considering the decrease in DED via product reformulation, or sugar reduction (1.19%), and decrease in DED via product reformulation and sales reduction (1.07% and 0.94%, respectively, for counterfactual scenarios 2 and 3).

**Table 1:** Dietary energy density from sugar-sweetened beverages in 2016 (baseline) and 2018 (counterfactual scenarios), by age group.

Scenario and age group	Dietary energy density from sugar-sweetened beverages (%)					
	Mean	5th percentile	25th percentile	50th percentile	75th percentile	95^th^ percentile
**2016 (baseline)**						
Total	1.13	0.00	0.03	0.38	1.52	4.63
0 to <10 years	0.92	0.00	0.02	0.26	1.18	4.01
10 to <18 years	1.93	0.04	0.41	1.24	2.70	6.15
18 to <65 years	1.24	0.00	0.07	0.51	1.70	4.83
≥65 years	0.42	0.00	0.00	0.02	0.31	2.32
**2018 (product reformulation—counterfactual 1)**						
Total	1.09	0.00	0.03	0.37	1.47	4.48
0 to <10 years	0.89	0.00	0.02	0.25	1.14	3.88
10 to <18 years	1.87	0.03	0.39	1.19	2.60	5.96
18 to <65 years	1.20	0.00	0.06	0.49	1.64	4.67
≥65 years	0.41	0.00	0.00	0.02	0.30	2.24
**2018 (product reformulation and sales reduction—counterfactual 2)**						
Total	0.98	0.00	0.03	0.33	1.32	4.02
0 to <10 years	0.80	0.00	0.02	0.23	1.02	3.48
10 to <18 years	1.68	0.03	0.35	1.07	2.34	5.36
18 to <65 years	1.07	0.00	0.06	0.44	1.47	4.19
≥65 years	0.37	0.00	0.00	0.01	0.27	2.01
**2018 (product reformulation and sales reduction—counterfactual 3)**						
Total	0.86	0.00	0.02	0.29	1.16	3.54
0 to <10 years	0.71	0.00	0.02	0.20	0.90	3.06
10 to <18 years	1.48	0.03	0.31	0.94	2.06	4.71
18 to <65 years	0.95	0.00	0.05	0.39	1.30	3.69
≥65 years	0.32	0.00	0.00	0.01	0.23	1.77

Counterfactual 1 considers sugar reduction due to product reformulation; counterfactual 2 considers sugar reduction due to product reformulation and sales decrease, using market data adjusted by tax information; counterfactual 3 considers sugar reduction and sales decrease, using only tax information.

In Table 2, the PIF and number of cases of obesity prevented, by age group, were estimated for the first counterfactual considering only reformulation of SSBs, particularly sugar reduction. The impact fraction was higher in adolescents (0.012%), corresponding to a yearly number of cases of obesity prevented of 0.76. In adults aged 18 to <65 years, the PIF was 0.008%, with a higher number of prevented cases of obesity per year (6.83). When estimating the impact considering decreases of sugar content and of sales, using market data adjusted by tax revenue information (counterfactual 2) or only tax information (counterfactual 3), a similar tendency was observed. The PIF in adolescents was 0.045% and 0.089%, respectively, and the corresponding numbers of prevented cases of obesity per year were 2.93 and 5.82.

**Table 2:** Potential impact fraction (PIF) and prevented obesity cases due to the decrease in sugar content of sugar-sweetened beverages (counterfactual 1) and due to decreases in sugar content and consumption volume (counterfactuals 2 and 3).

Scenario and age group	Cohort study	Obesity incidence/100 persons/year (95% CI)	National population size*	PIF (%)	Prevented obesity cases per year
**Product reformulation (counterfactual1)**					
0 to <10 years	GXXI	1.76 (1.62, 1.90)	913,211	0.006%	0.98
10 to <18 years	EPITeen	0.61 (0.43, 0.83)	1,076,983	0.012%	0.76
18 to <65 years	EPIPorto	1.36 (1.06, 1.72)	6,115,151	0.008%	6.83
≥65 years	EPIPorto	2.62 (1.82, 3.61)	2,194,959	0.003%	1.83
Total					10.39
**Product reformulation and sales reduction (counterfactual 2)**					
0 to <10 years	GXXI	1.76 (1.62, 1.90)	913,211	0.024%	3.91
10 to <18 years	EPITeen	0.61 (0.43, 0.83)	1,076,983	0.045%	2.93
18 to <65 years	EPIPorto	1.36 (1.06, 1.72)	6,115,151	0.032%	26.24
≥65 years	EPIPorto	2.62 (1.82, 3.61)	2,194,959	0.013%	7.29
Total					40.37
**Product reformulation and sales reduction (counterfactual 3)**					
0 to <10 years	GXXI	1.76 (1.62. 1.90)	913,211	0.049%	7.82
10 to <18 years	EPITeen	0.61 (0.43. 0.83)	1,076,983	0.089%	5.82
18 to <65 years	EPIPorto	1.36 (1.06. 1.72)	6,115,151	0.062%	51.35
≥65 years	EPIPorto	2.62 (1.82. 3.61)	2,194,959	0.023%	13.34
Total					78.33

Counterfactual 1 considers sugar reduction due to product reformulation; counterfactual 2 considers sugar reduction due to product reformulation and sales decrease, using market data adjusted by tax information; counterfactual 3 considers sugar reduction and sales decrease, using only tax information.

Considering the final scenario (counterfactual 3), the next highest impact fraction, after that in the adolescent age group, was observed in adults (0.062%), and then in children (0.049%). Older adults (aged ≥65 years) showed the lowest projected impact (0.023%).

## Discussion

Our data show that the SSB levy in Portugal achieved its goal by decreasing sales of SSBs and incentivizing reformulation towards lower levels of sugar content. Our modelling analyses estimate that the Portuguese sugar tax will have a positive effect on the population’s incidence of obesity in the medium to long term. The reduction in sales of SSBs was estimated at 6.6 million litres per year (21% decrease in volume), and the average SSB energy density decreased by 3.1 kcal per 100 ml. The projected impact of such a fiscal intervention on health outcomes, including obesity, depends on various factors such as obesity prevalence, the level of consumption of SSBs, the magnitude of the tax, the initial baseline tax, and the price of these products. Taking into account the observed market changes and consequent reduction in sugar intake due to SSBs, we have estimated that in the medium term the tax-related reduction in sugar consumption will prevent around 40–78 new cases of obesity in Portugal every year. Even though the projected impact of the tax (mediated through reformulation and reduced consumption) may seem small, it is actually 4–8 times larger than would be observed from reformulation alone.

Analysing the fraction of dietary energy that comes from SSBs, we notice that the age group where these drinks contribute the most to energy intake is people 10 to <18 years of age. For this age group, we also observed the most pronounced reduction of obesity incidence. This group had the lowest baseline incidence of obesity but the highest fraction of dietary energy from SSBs. The projected impact was lower for the 2 groups with the highest baseline incidence of obesity but low baseline intake of SSBs (0–10 years and ≥65 years).

### Comparison with other research

There is substantial methodological diversity in studies assessing the effectiveness (pre- and post-tax) of levies introduced on SSBs in different countries. Most studies evaluating the effectiveness of a sugar tax focus on sales and price changes [32–38]. Studies from the US on adults, children, and adolescents used SSB sales data combined with data from the National Health and Nutrition Examination Survey, which includes a direct measurement of BMI. In addition, they assess the change in calories in the diet from SSBs [29,39]. Another study from Mexico translated sales data into calories combined with BMI and used these data to assess the impact Mexico’s sugar tax had on obesity prevalence in the population [40]. We are not aware of any other study that measured tax-triggered reduction of sugar intake and related it to obesity outcomes on a national scale.

A meta-analysis published in 2013 showed mixed results of sugar taxes, with taxes decreasing obesity indicators in 6 eligible studies, whilst increasing them in 2 studies [9]. A 2013 study from the UK, modelling a 20% tax, predicted a 1.3% decrease in obesity prevalence and 0.9% decrease in overweight prevalence. Similarly to our study, the effect was most pronounced for younger age groups [11]. The UK study was conducted before sugar tax implementation, and the figures following this event are still unknown. A more recent review from 2016 [41] examining sugar tax effectiveness in combating the obesity burden in middle-income countries included 3 studies: a quasi-experimental study from Peru and 2 modelling studies, 1 from India and 1 from South Africa. While the modelling studies showed a 3% decrease in obesity and overweight prevalence, the quasi-experimental study from Peru showed an increase of 0.9%. Unlike the modelling study, the Peruvian sample was not representative of the whole population and only included 19- to 49-year-old women. In the Peruvian study, the tax applied was 10% (c.f. 20% for the 2 modelling studies), and it has been previously shown that for such a tax to be effective it should be set at >20% [42]. These differences could account for at least some of the discrepancies in the findings.

A German modelling study showed that the implementation of a 20% tax on SSBs would lead to a decrease in obesity and overweight of 3% and 4%, respectively, with the largest benefit being projected for 20- to 29-year-old males [43].

In line with our study, a study from Mexico modelled the predicted change in obesity using a pre- and post-tax comparison of caloric intake from SBBs. It estimated that 10 years after tax implementation (in 2014), obesity prevalence would fall by 2.54%, with the biggest impact for those between 20 and 35 years of age [40].

One of the countries where the application of a levy on SSBs is currently being discussed is Australia. A study modelling the potential health benefits of a 20% tax in this country showed the impact on weight loss across the population, again with young people benefiting the most [41].

Our findings estimate a lower effect of the SSB tax on obesity in Portugal than predicted by modelling studies performed in other countries. This difference may be related to the price elasticity of demand (PED). Previous studies have shown that the PED values for foods are very low, and regarding SSBs, a 20% price increase is needed to have a potential impact of price changes on demand. In Portugal, the price increase in SSBs as a result of the tax was below this threshold. According to Santos [44], an analysis of SSB average prices in Portugal between 2016 and 2017 showed a statistically significant increase in SSB average prices, but of only 18%. According to this study, the average price of SSBs increased from 1.1€/litre in 2016 to 1.3€/litre in 2017 (+0.2€/litre). Moreover, another study of the price increase of SSBs in Portugal, covering a larger period of analysis (between February 2015 and January 2018) and providing disaggregated data according to the SSB levy tier, found similar results. Average price increases of about 16% and 19% were found for the drinks with sugar contents of ≥80 grams/litre and <80 grams/litre, respectively [45].

### Strengths and limitations

In the light of the ongoing political discussion about the effectiveness of fiscal interventions in public health, monitoring and reporting the impact of such measures is crucial. The main strength of our study lies in the consideration of the population-wide change in sugar consumption as a result of both the decrease in volume of SBBs sold and their reformulation. This allowed the modelling of the changes in obesity incidence across various age groups in Portugal. Another strength of the study is the use of individual consumption data from a national representative sample that allowed generation of specific estimates of the impact in the different age groups. This would not have been possible if only market data were used.

In epidemiological studies it is difficult to measure a direct and observable effect of a change to a risk factor on prevented cases of disease. In the case of the sugar tax, the challenge is to show that the new cases prevented can be attributed to the levy, independently of other changes in the environment. The model used, PIF, and similar modelling tools used frequently in assessing population-wide interventions, allow estimation of the reduction in obesity attributable to the decreased availability and consumption of sugar through SSBs.

The analyses include data from various sources. As such, they can accumulate bias related to the methodological differences between these sources. As some of these datasets were incomplete, data had to be imputed. However, imputation was performed for less than 20% of the cases. Estimates for sales, and therefore availability of sugar from SSBs, in the Portuguese market were different depending on the source. The reduction in sales, and consequent prevented new cases of obesity, estimated using the tax payment data source was higher than that obtained using the Nielsen market data, even after calibration. Despite this, changes in market data and tax data were consistent with each other, and the market data were consistent with the information on individuals’ consumption.

Since it was not possible to know from the retail sales data the trend by age, the same trend was assumed for all age groups. Using the individual consumption from the national dietary survey as baseline, we estimated the trend in consumption assuming the proportional trend reduction observed in retail sales data. We only used information on non-concentrated drinks since this is the information available in the tax data for 2017 and in the retail sales data.

Another limitation is lack of consideration of the substitution effect in our models. As some part of SSB consumption could shift towards the consumption of other drinks containing calories but not subject to the tax, such as fruit juices or milk drinks, the projected impact on obesity incidence might be overestimated.

Since it was not possible to assess to what extent individual SSB consumption was substituted by consumption of other types of beverage (juice, water, milk, or even alcoholic beverages), we assumed that a decrease in SSB consumption implied a substitution with water.

### Policy implications

Our study provides strong evidence that the Portuguese SSB tax was associated with a reduction in the DED related to SSB consumption as a result of a decrease in sales and beverage reformulation. The reduction in sales (presumably due to price increases) led to a much greater impact on obesity reduction than reformulation alone. This adds to a body of evidence suggesting that fiscal interventions are more effective than voluntary industry measures [46].

We estimate that the Portuguese SSB tax will prevent fewer than 78 cases of obesity per year. Given the scale of the obesity problem this is worthwhile; however, we note that fiscal measures are not a panacea and should complement a panoply of other measures to address this complex system problem.

### Conclusion

The example from Portugal adds to evidence that taxation of SSBs is effective in decreasing average energy and sugar content of drinks as well as consumption. Since younger individuals tend to be the heaviest consumers of SSBs, taxation policies are most likely to protect this age group from becoming obese. The levy on SSBs caused a significant acceleration in the reduction of SSB sales and sugar intake. Reformulation alone, without the decrease in sales, would have a far smaller effect on obesity incidence in the Portuguese population.

Despite taxation policies becoming more and more popular as public health tools, their isolated impact is limited. Thus, continuous and integrated multisectoral interventions focused on improving dietary habits should be a priority for governments in combating the epidemics of non-communicable diseases.

## Supporting information

S1 STROBE Checklist (DOCX)

## Abbreviations

DED: dietary energy density
IAN-AF 2015–2016: National Food, Nutrition and Physical Activity Survey 2015–2016
OR: odds ratio
PIF: potential impact fraction
PRBEB: Portuguese Association of Non-Alcoholic Drinks
RR: relative risk
SSB: sugar-sweetened beverage
